# Understanding the molecular mechanisms underlying graft success in grapevine

**DOI:** 10.1186/s12870-019-1967-8

**Published:** 2019-09-11

**Authors:** M. Assunção, C. Santos, J. Brazão, J. E. Eiras-Dias, P. Fevereiro

**Affiliations:** 1 Plant Cell Biotechnology Laboratory, Instituto de Tecnologia Química e Biológica António Xavier (Green-it Unit), Universidade Nova de Lisboa, Apartado 127, 2781-901 Oeiras, Portugal; 2 Genetics and Genomics of Plant Complex Traits (PlantX) Laboratory, Instituto de Tecnologia Química e Biológica António Xavier (Green-it Unit), Universidade Nova de Lisboa, Apartado 127, 2781-901 Oeiras, Portugal; 3 Instituto Nacional de Investigação Agrária e Veterinária (Biotechnology and Genetic Genetic Resources Unit) INIAV-Dois Portos, Quinta da Almoínha, 2565-191 Dois Portos, Portugal; 4 Departamento de Biologia Vegetal, Faculdade de Ciências da Universidade de Lisboa, Campo Grande, 1749-016 Lisboa, Portugal

**Keywords:** Grapevine, Grafting, Graft compatibility, Molecular mechanism of grafting, Vascular differentiation, Transcriptional regulation of grafting, Post-transcriptional regulation of grafting

## Abstract

**Background:**

Grafting is an intensive commercial practice required to protect the European grapevine against the Phylloxera pest. Rootstocks resistant to this pest are hybrids of American vine species with different levels of compatibility with European *Vitis vinifera* varieties. Aiming to understand what drives grafting compatibility in grapevine, a transcriptomic approach was used to search for master regulators of graft success. Two scion/rootstock combinations, with different levels of compatibility, were compared in a nursery-grafting context at two stages, at 21 and 80 days after grafting.

**Results:**

In the most compatible combination, an earlier and higher expression of genes signaling the metabolic and hormonal pathways as well as a reduced expression of genes of the phenolic metabolism and of the oxidative stress response was observed. At 80 days after grafting a higher expression of transcription factors regulating vascular maintenance, differentiation and proliferation was obtained in the most compatible combination. Moreover, lower expression levels of microRNAs potentially targeting important transcription factors related to plant development was observed in the more compatible combination when compared to the less compatible one.

**Conclusion:**

In this context, a set of regulators was selected as potential expression markers for early prediction of a compatible grafting.

**Electronic supplementary material:**

The online version of this article (10.1186/s12870-019-1967-8) contains supplementary material, which is available to authorized users.

## Background

Grafting is a very ancient method used worldwide for clonal propagation, to reduce juvenility and to overcome many biotic and abiotic stresses. Nowadays it is a widespread technique used in fruit trees, vegetables and flower production and therefore with an enormous agricultural and economic impact [1]. In the European viticulture, grafting is almost imperative due to phyloxera, an insect that feeds from the roots of *Vitis vinifera* cultivars leading vines to death. So far, the only way to overcome this pest has been to graft the European cultivars in American or American hybrid resistant rootstocks [2]. Grapevine grafting may be subjected to incompatibility since two genetically different entities are put together. Graft incompatibility may be defined as the failure to form a successful graft union between two plant parts, i.e. the fail to form a proper functional composite plant when all other requirements, such as technique, timing, phytosanitary and environmental conditions are satisfied. Incompatibility may be expressed even after many years of normal growth, not only in grapevine but also in other species such as pear-quince, apricot, and pear [3–5]. Most of the incompatibility studies were directed to morphological and physiological observations between compatible and incompatible unions [6, 7]. The development of a graft union starts with the healing of the graft zone by a wound response where the living regions of the scion and stock in contact initiate the proliferation of parenchymatous cells that origin a mass of undifferentiated cells called callus. This callus works as a bridge between the two plant parts until differentiation of the new cambial cells into xylem and phloem tissues, enabling the vascular connection between scion and rootstock [6, 8].

Early detection of graft incompatibility is of great importance for nurseries and farmers since it could be used as a tool for early selection of best graft combinations. Many detection methods have been developed such as in vitro techniques [3], histological observation [9, 10] isozyme analyzes [11, 12] and phenolic analyzes [4, 13–15]. These are important methods, however these phenologic and metabolic markers vary a lot depending on the grafting combinations, environmental and soil conditions, making graft incompatibility detection very difficult to perform. Only recently the molecular mechanisms associated with grafting began to be studied. In a broad approach, Cookson et al. [16] studied the transcriptional profile of graft union in grapevine autografts. They found genes differentially expressed between 3 and 28 days after grafting to be related to cell wall modification, wounding, hormone signaling and secondary metabolism. In a later study, Cookson and collaborators also compared the gene expression profile at the graft union of heterografts (rootstock and scion from different genotypes) with autografts (rootstock and scion from the same genotype) and found up-regulation of genes involved in stress responses, suggesting that it could be related to the detection of a non-self grafting partner [17]. Irisarri et al. [18] detected a higher accumulation of antioxidant gene transcripts in compatible grafts early in graft development of pear/quince and suggested to be associated with better protection of the damaged tissues. Melnyk et al. [19] reported that in *Arabidopsis* grafts the xylem is formed only after the phloem connection, and for the phloem connection a group of auxin response factors act below the graft junction, such as AXR1 and ALF4. Interestingly, mutating *AXR1* in the upper side of the graft union rescued the phloem connection in a rootstock mutant of *AXR1* [19]. These recent findings attribute an important role in the spatial and temporal communication between scion and rootstock to the process of graft connection. Chen et al. 2017 analyzed the transcriptome of *Litchi* compatible autograft and incompatible heterograft at 2 h, 4 days and 21 days after grafting. The results suggested that genes expression related to wound response, auxin (IAA) and signal transduction pathways could have a key role in *Litchi* grafting healing process. More recently, the important role of auxin was also reported in *Citrus* compatibility studies. [20] Although the recent findings, the molecular mechanisms of grafting are still largely unknown, particularly in woody plants.

By studying and understanding the molecular mechanisms of the union formation in grapevine we will be closer to develop molecular markers for compatibility, less variable and more suitable for breeding. Having that in mind we compared two heterografts of two clones of the cultivar Touriga Nacional grafted in the rootstock Richter110 which showed different levels of compatibility. Touriga Nacional is presently the main grapevine cultivar used to produce wine in Portugal and it is known to produce the best quality Portuguese wines. Richter 110 (110R), a worldwide used rootstocks, has shown to have deficient compatibility with Syrah cultivar [21] and reported to have different levels of graft compatibility with Touriga Nacional (TN). We explored, in a nursery-grafting context, two different time points; 21 days after grafting, when grafts are taken out of the callus induce chamber to be transferred to the field and, 80 days after grafting, i.e. after 2 months in the field and when the root system is already developed. By looking at the differentially expressed genes and micro RNAs (miRNA)s between the more and the less compatible combination we aimed not only to unveil what mediates graft compatibility but also to find master regulators of graft union formation that could be used as molecular markers for early prediction of graft success in grapevine.

## Results

### Analysis of grafting success

**Fig. 1:**
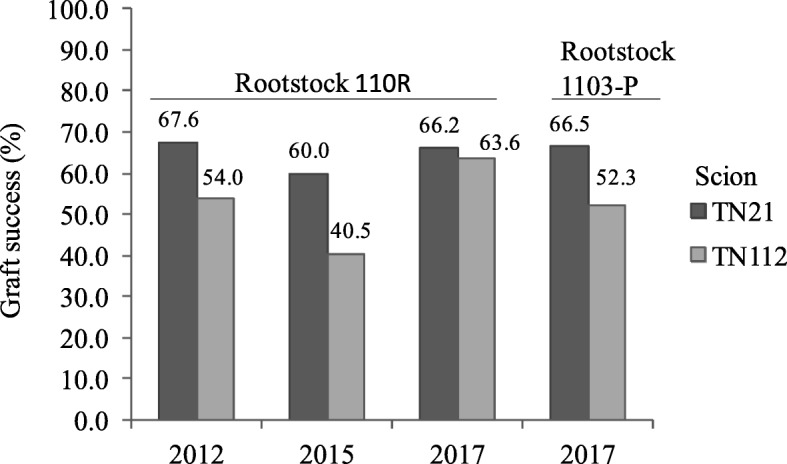
Percentage of graft success in the heterografts. Graft success was evaluated at the end of the vegetative cycle for Touriga National clones TN21 and TN112 grafted on the rootstock 110R, in 2012, 2015 and 2017, and for the same clones grafted in 1103-P in 2017

The graft compatibility of the combinations TN21/110R and TN112/110R was accessed quantifying the graft success, i.e. counting the number of grafts with well-developed root and shoot system and with a well-established union at the end of the vegetative cycle. Three independent trials, performed in independent years, revealed TN21/110R combination was more successful than TN112/110R (Fig. 1). Touriga Nacional grafted on the rootstock 1103-P was also evaluated, and TN21 proved again to have a higher graft success (66.5%) when compared with TN112 (52.3%).

The mean comparison of the graft success over 3 years of clones TN21 and TN112 over 110R revealed a *p*-value of 0.169. When adding the comparison of the graft success obtained with 1103-P, the p-value retrieved was of 0.051. Based on this, it was considered that there is a difference in graft compatibility in between the two clones being the TN21/110R combination the more compatible and TN112/110R the less compatible one.

To study the molecular mechanisms responsible for these different compatibility levels, samples were collected 21 and 80 days after grafting (DAG). At 21DAG, few unsuccessful or null grafts where detected, characterized at this time point by the absence of callus or the presence of dead tissue in the area of the scion and rootstock in contact. At this stage, there is no incompatibility per se, the formation of the callus is a consequence of the response to the wound and to the phytohormones applied in the wax during the graft procedure (see Methods section), and there is still no interaction of the two vascular systems. Incompatibility is only detectable after the grafts have been transferred to the field. At 80DAG, the majority of unsuccessful grafts were observed, i.e. grafts that failed to form a union between two plant parts at graft interface (Additional file 1).

**Fig. 2:**
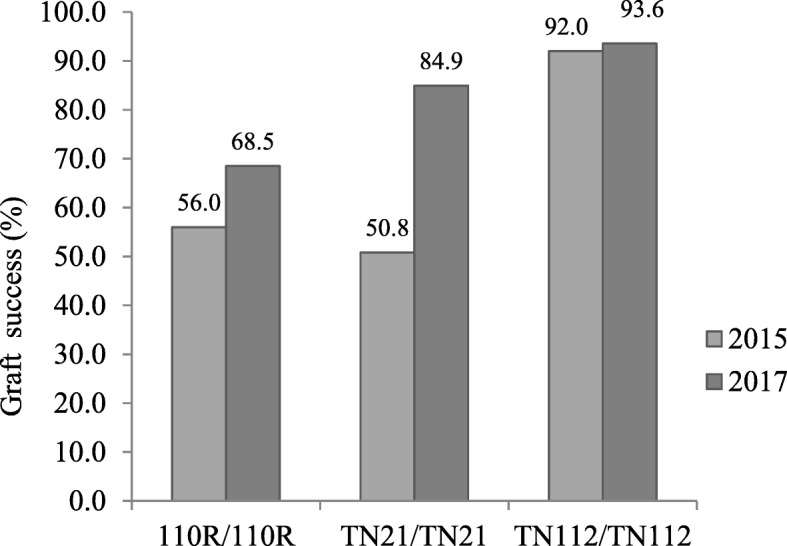
Percentage of graft success in the autografts. Graft success was evaluated at the end of the vegetative cycle for autografts of the rootstock 110R (110R/110R) and the scions TN21 (TN21/TN21), and TN112 (TN112/ TN112), in 2015 and 2017

Autografts are usually reported to be compatible since it is expected no incompatibility when a genotype is grafted onto itself. Autografts of the Touriga Nacional clones and of the 110R rootstock were evaluated in the second and third field trial (Fig. 2). In 2015, the two autografts of Touriga Nacional had different graft success rates, with the autograft TN112/TN112 exhibiting a success rate of 92% while TN21/TN21 had only 50% of success and the 110R/110R only 56%. In 2017 success rates were comparably higher than in 2015, with TN112/TN112 showing, as in the previous trial, the highest compatibility rate. These results led to the exclusion of autografts as compatible controls in the transcriptomic approach.

### Genes differentially expressed between the two heterografts at 21DAG

The transcriptome analysis revealed 33 differentially expressed genes (DEG) between the more compatible combination (TN21/110R) and the less compatible one (TN112/110R) at 21DAG. In Table 1 it is shown the DEG at 21 DAG, where 17 transcripts are up-regulated (more abundant) in the more compatible combination (TN21/110R) and 16 are down-regulated in the more compatible when compared to the less compatible combination (TN112/110R).

**Table 1:**
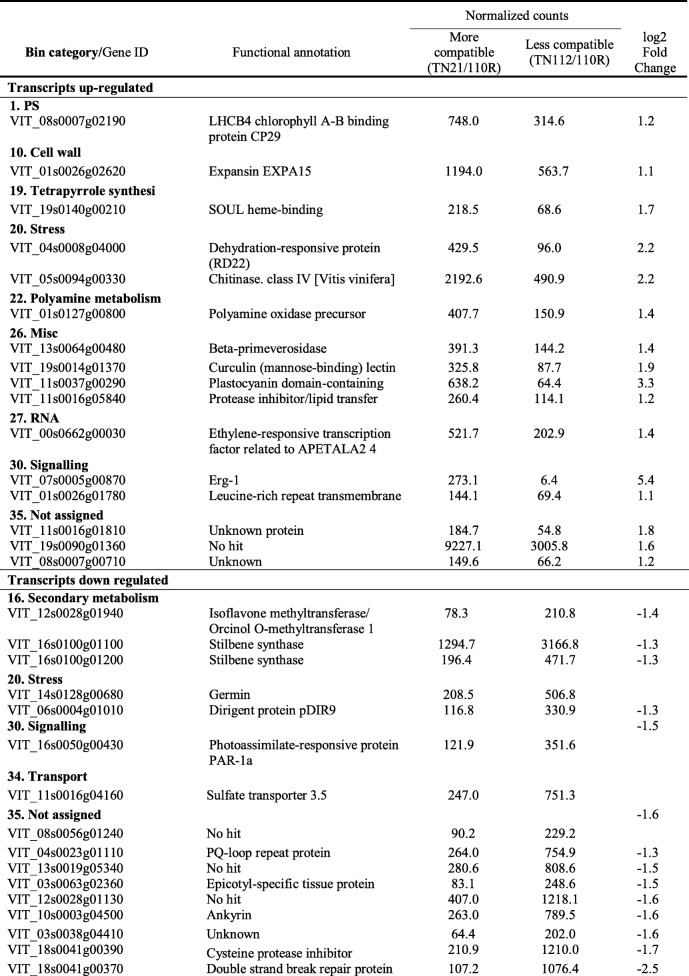
Differentially expressed genes at 21 DAG between the more compatible (TN21/110R) and the less compatible (TN112/110R) combination

Transcripts belonging to the BIN categories “Photosysthesis”, “Cell wall”, “Tetrapyrrole synthesis”, “Polyamine metabolism”, “Miscellaneous”, “RNA” and “DNA” and “Signaling” were found to be more expressed in the more compatible combination (TN21/110R) at 21DAG.

From the genes more expressed in the more compatible combination it was found an expansin gene (VIT_01s0026g02620), codifying for a protein that responds to auxin signaling, involved in cell wall loosening and cell elongation in acidic growth [22]; an ethylene responsive transcription factor, (VIT_00s0662g00030), member of the AP2/ERF TF superfamily known to be an important regulator of developmental processes and to responses of various types of biotic and environmental stresses [23]; and a EP3 chitinase (VIT_05s0094g00330).

The category “DNA” comprises a histone H2A.6 (VIT_00s0868g00020) up-regulated in the more compatible heterograft and the category “signaling” contains three transcripts, two differentially more expressed in the TN21/110R heterograft: one protein kinase, one ethylene response factors, Erg-1 (VIT_07s0005g00870), with a BIN description of signaling in sugar and nutrient physiology. This ethylene response factor has a log_2_ fold change of 5.4, the highest of this analysis. Contrastingly, three genes codifying enzymes of the phenylpropanoids: two stilbene synthases, (VIT_16s0100g01100, VIT_16s0100g01200) directly involved in the synthesis of resveratrol and one Orcinol O-methyltransferase1 (VIT_12s0028g01940), which are more expressed in the less compatible combination (TN112/110R).

### Genes differentially expressed between the two heterografts at 80DAG

The transcriptome analysis revealed 63 DEGs between the two heterografts at 80DAG. In Table 2, it can be seen the DEG, 26 of which are up-regulated and 37 down-regulated in the more compatible combination (TN21/110R) in comparison to the less compatible one.

**Table 2:**
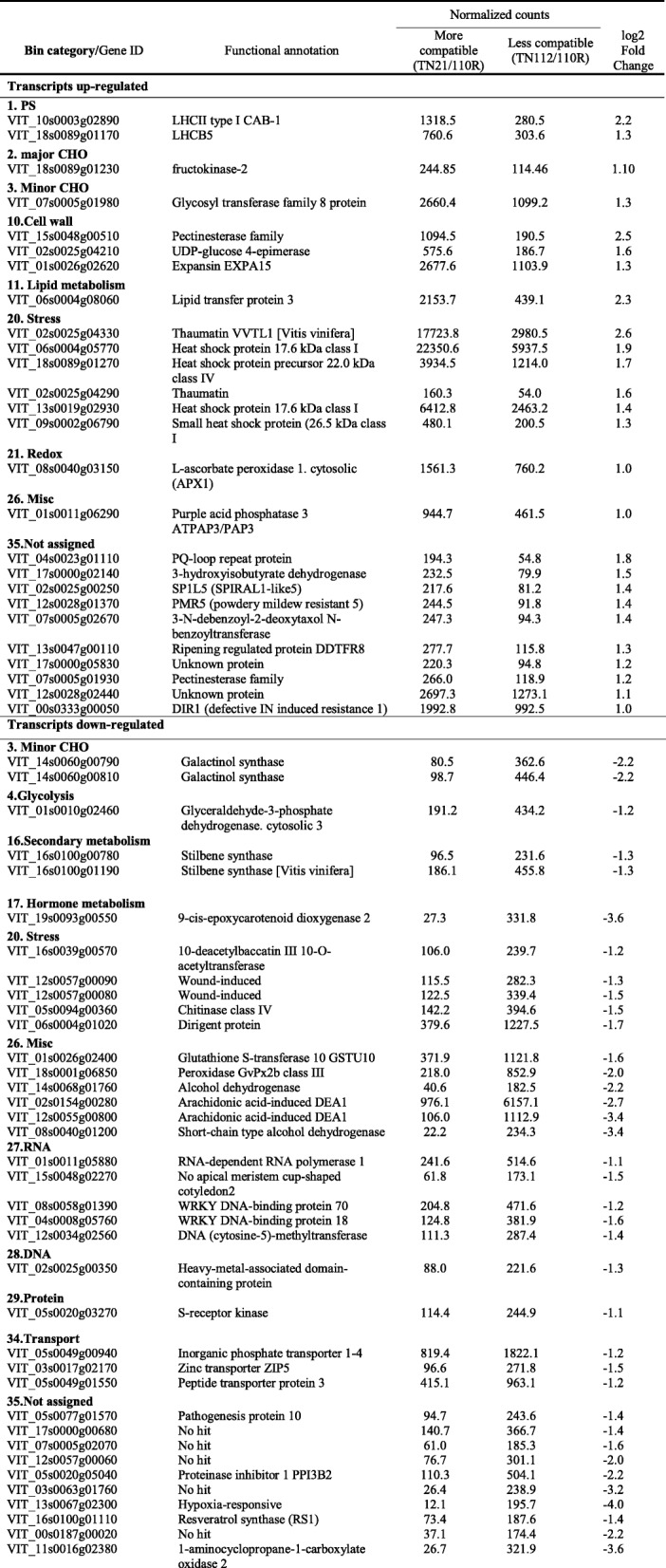
Differentially expressed genes at 80DAG between the the more compatible (TN21/110R) and the less compatible (TN112/110R) combination

The more compatible combination (TN21/110R) showed a higher abundance of transcripts associated with cellular processes, like the categories “Photosynthesis”, “Major CHO” and “Cell wall”. This last category comprises three transcripts: a pectin methylesterase (VIT_02s0025g04210), a UDP-glucose 4-epimerase (VIT_15s0048g00510) and an expansin (EXPA15, VIT_01s0026g02620).

Regarding the less compatible combination, DEGs belonging to categories “RNA”, “Secondary metabolism”, “Minor CHO metabolism”, and “Miscellaneous” were found to be more expressed in the less compatible combination (TN112/110R). The category “RNA” included three TFs, a NAC (VIT_15s0048g02270) and two WRKYs, WRKY18 (VIT_04s0008g05760) and WRKY70 (VIT_08s0058g01390), as well as an RNA-dependent RNA polymerase 1 (RDR1, VIT_01s0011g05880) and a DNA methyltransferase (VIT_12s0034g02560). WRKY TFs play a dual role in the brassinosteroids-mediated regulation of PAMP (pathogen-associated molecular patterns)-triggered immunity (PTI) signaling [24]. In the category “secondary metabolism”, two stilbene synthases (VIT_16s0100g01190, VIT_16s0100g00780) were also more expressed in the less compatible combination (TN112/110R). A resveratrol synthase (VIT_16s0100g01110) was also more expressed in the less compatible combination. Stilbene synthases are precursors of resveratrol, which have been related to the initiation of the hypersensitive response in *Vitis*, and related to programmed cell death (PCD) [25].

Within the category “Miscellaneous” two transcripts highly expressed in the less compatible combination annotated as Arachidonic acid-induced DEA1 (VIT_12s0055g00800 and VIT_02s0154g00280) were found. Still in this category, transcripts coding antioxidant proteins were found all more abundant in the less compatible combination, like two short chain dehydrogenase, SDR (VIT_14s0068g01760, VIT_08s0040g01200), a glutathione S-transferase (VIT_08s0040g01200), a peroxidase (VIT_18s0001g06850), and two lipid transfer protein (VIT_02s0154g00280, VIT_12s0055g00800).

### Transcriptome profiling between 21DAG and 80DAG

**Fig. 3:**
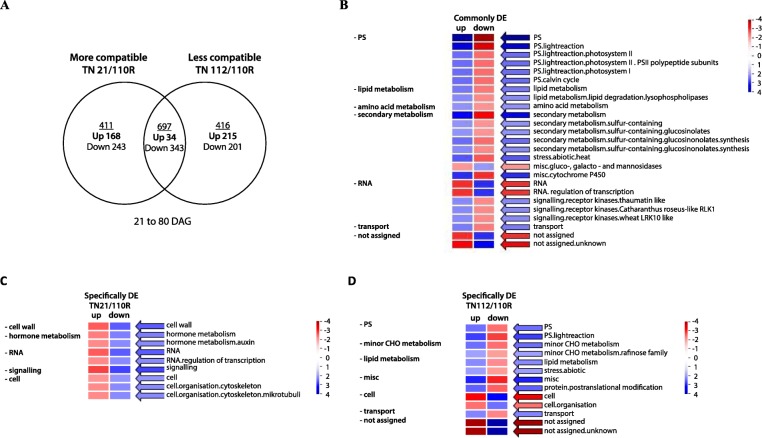
DEGs between 21DAG and 80DAG found in both heterografts. DEGs with significant differences were selected based on a FDR < 0.05, a log2 fold change > 1 and more than 100 counts. Venn Diagram with the number of DEGs found between 21DAG to 80DAG in the more compatible heterograft (TN 21/110R) and in the less compatible one (TN 112/110R) (**a**). PageMan visualization of MapMan functional categories enriched in the DEG found commonly DE (TN21/110R and TN21/110R) (**b**), specifically DE in the more compatible (TN21/110R) (**c**) and specifically DE in the less compatible (TN112/110R) (**d**). The over representation/under representation of the functional categories in the up/down-regulated genes is given by shades of blue and red, respectively

Transcriptome analysis was also performed between time points (from 21DAG to 80DAG) for each combination. A total of 697 genes were commonly DE in both heterografts (Fig. 3), whereas 411 DEGs were only DE in the more compatible combination (TN21/110R) and 416 only in the less compatible combination (TN112/110R) (Fig. 3a).

The functional categories enrichment of the common DEG from 21 to 80DAG (Fig. 3 b) showed an over-representation of the up-regulation on the transcripts “Photosynthesis”, “Lipid metabolism”, the subcategory “signaling .receptor kinases”, “amino acid metabolism”, “secondary metabolism”, the subcategory “Stress.abiotic.heat”, “Transport” and Miscellaneous subcategory “misc.gluco-,galacto and manosidases”. Oppositely there is an over-representation of the down-regulation of the categories “RNA”, the Miscellaneous subcategory “misc.cytochrome P450” as well as the “not assigned” category.

The categories “PS”, “minor CHO metabolism”, “lipid metabolism”, “stress. Abiotic”, “protein” and “transport” were enrich from 21 to 80DAG in the less compatible TN112/110R combination (Fig. 3d). In the more compatible combination (TN21/110R) there is an enrichment of down-regulated transcripts of the categories “Cell Wall”, “hormone metabolism”, “RNA”, “signaling” and “Cell” from 21 to 80DAG (Fig. 3c).

**Fig. 4:**
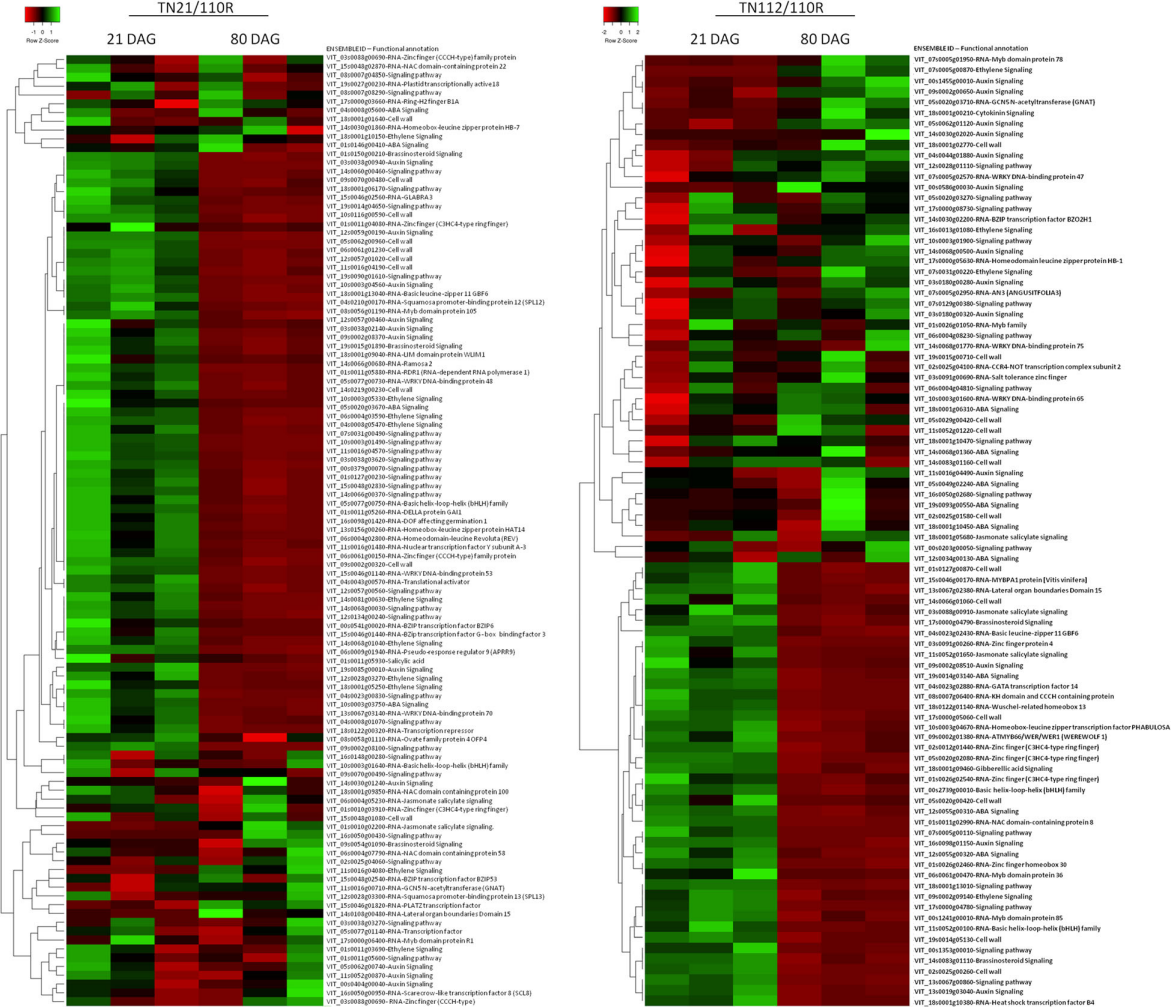
Heatmap of transcripts involved in hormone signaling, pathway signaling, TF and cell wall functions found differentially expressed between time points (21DAG and 80DAG) for each combination**.** A total of 108 DEGs in TN21/110R and a total of 90 DEG in TN112/110R were clustered using Kendall’s Tau distance and an average linkage

Due to the importance of these categories in the graft healing process and in the vascular differentiation, these transcripts are presented as a heatmap for both combinations(Fig. 4). For this, some transcripts annotated by Grimplet et al. [26], as belonging to the categories under analysis, were rescued from the data sets and added to those obtained by the BIN categorization. The clustering of this group of transcripts, using an average linkage clustering method with Kendall’s Tau distance measurement, revealed the difference in the expression timing between combinations, where a higher number of these genes was more expressed at 21DAG in the more compatible combination. When clustering by categories and subcategories the same group of genes (Additional file 2), and looking at the expression in all libraries it is possible to see, despite the same profile of up/down regulation, the differences in the expression level. In the specific DEG of the more compatible combination (TN21/110R), transcripts of the auxin and ethylene signaling pathways are more expressed at 21DAG. Of the 10 transcripts involved in the auxin signaling pathway, seven were more abundant at 21DAG and only three are up-regulated at 80DAG. For the ethylene signaling pathway, the same pattern is observed, seven transcripts being more abundant at 21DAG, and only three up-regulated at 80DAG. In contrast, there was a higher expression at 80DAG of most of the transcripts from the signaling pathway of these two phytohormones in the less compatible combination (TN112/110R). Specifically, DEG in this combination, 10 out of 13 transcripts of the auxin signaling pathway are more expressed at 80DAG, while from four ethylene signaling related transcripts, three are up-regulated 80DAG. Furthermore, more transcripts related to the stress hormone abscisic acid (ABA) are differentially expressed in the less compatible combination, being six out of nine genes overexpressed in the less compatible combination at 80DAG.

In total, 138 TFs were differentially expressed from 21 to 80DAG in both combinations, 57 commonly present in both combinations, 42 exclusively in the TN21/110R and 39 exclusively in the T112/110R combination. Some of these TFs are directly involved in the maintenance and differentiation of vascular cambium cells, such as VIT_19s0027g01120 (LBD4), VIT_18s0001g10160 (WOX4) and VIT_18s0001g06430 (ATHB-6) [27–29].

TFs regulating auxin and ethylene pathways were also differentially expressed between time points, as well as, diverse MYB, WRKY, and C2H2 zinc finger TFs family members.

### Differential expression of transcription factors involved in the regulation of vascular differentiation

Due to the importance of the vascular tissue connection in the development of a successful graft, detailed expression quantification was undertaken between the two combinations and between the two time points on known regulators (TFs) of vascular differentiation (Table 3).

**Table 3:** Gene ID, functional annotation, description and function of the eight transcription factors analyzed by dPCR

Emsemble Genomes ID	Annotation Predicted (NCBI release 101)	Description	Function	References
VIT_19s0027g01120	Lateral organ boundaries protein 4 (LBD4)	LOB family protein	Cell proliferation in the cambium. Activation of phloem differentiation.	Yordanov et al. [28]; Guerriero et al. [29].
VIT_18s0001g10160	Wuschel homeobox 4 (WOX4)	Homeobox transcription factor	Stem cell maintenance in cambium and differentiation and/or maintenance of the vascular cambium.	Hirakawa et al. [30]; Suer et al. [31]; Guerriero et al. [29].
VIT_18s0001g06430	Homeobox-leucine zipper protein ATHB-6 (ATHB6)	b-ZIP transcriptional factor	Negative regulator of Abscisic acid signaling (ABA) pathway. Cell division and/or differentiation in developing organs.	Sӧderman et al. [27].
VIT_06s0004g03130	Auxin response factor 4 (ARF4)	Auxin response factor	Auxin signaling. Organ polarity, vascular development and organ asymmetry establishment.	Pekker et al. [32]; Hunter et al. [33].
VIT_07s0141g00290	Auxin-responsive protein IAA16-like (IAA16)	Aux/IAA family	Auxin signaling. Repressor of ARF response. Plant growth.	Korasick et al. [34]; Rinaldi et al. [35].
VIT_18s0001g02540	Response regulator ARR9	Type A ARRs	Negative regulation of cytokinin signaling. Callus and lateral root formation.	Perianez-Rodriguez et al. [36].
VIT_04s0008g06000	Ethylene-responsive transcription factor ERF3	Ethylene response factor AP2/ERF	Ethylene signaling. Cell division in developing vascular tissue. Xylem development.	Etchells et al. [37] Vahala et al. [38]
VIT_16s0013g00890	Ethylene-responsive element binding factor - ERF1	Ethylene response factor	Ethylene signalling. Cell division in developing vascular tissue.	Etchells et al. [37]

**Fig. 5:**
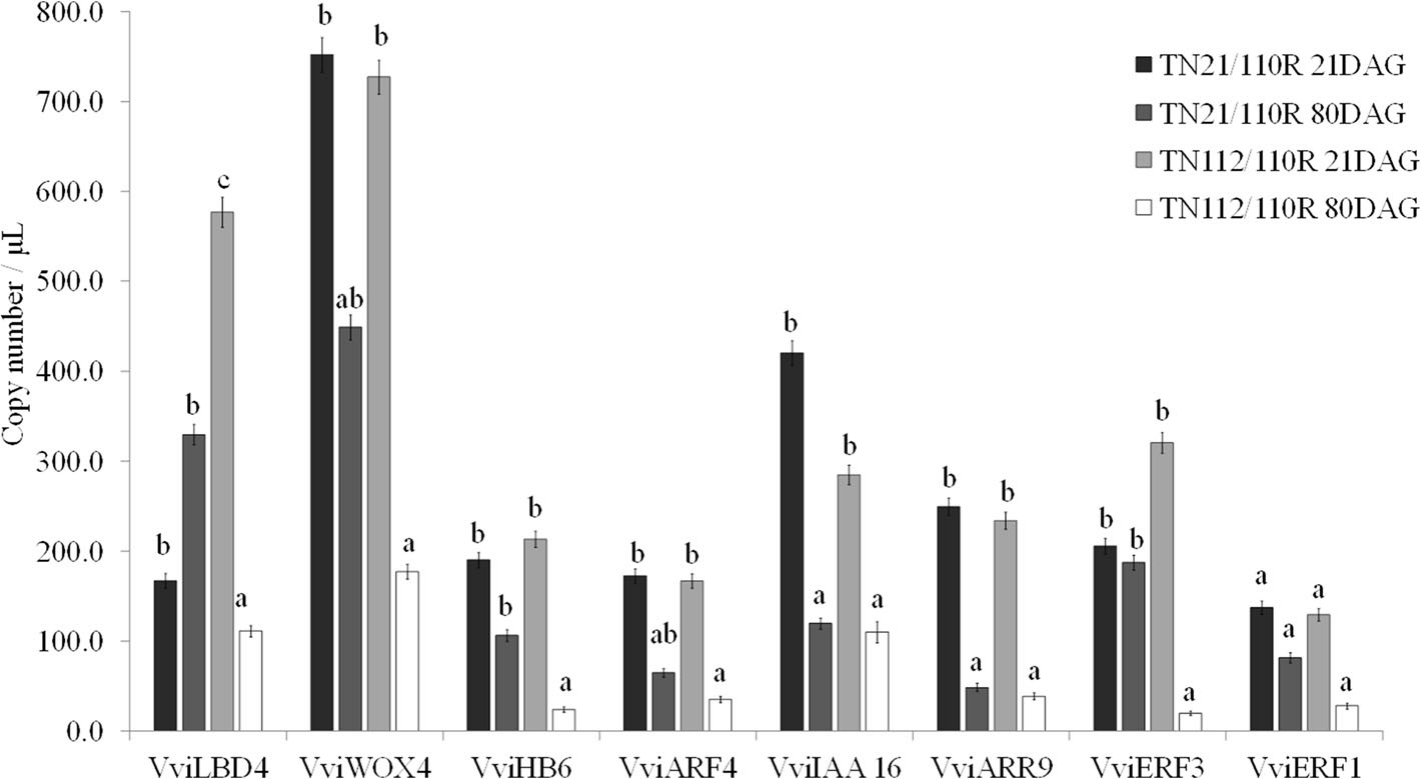
Quantification of TFs levels in the graft union tissue by dPCR. The gene expression level of eight TF was performed by dPCR, at the graft union tissue in the TN21/110R (more compatible) and TN112/110R (less compatible) heterografts in two time points, 21 days after grafting (DAG) and 80DAG. Error bars represent the confidence interval and different letters represent significant different values (*p* < 0.05) according to Mann-Whitney test

The expression of these eight TFs, previously found in the transcriptome data with significant differences in between 21 and 80DAG in both TN21/110R and TN112/110R but with no significant differences between them, was quantified by dPCR (Fig. 5).

As observed in MACE-Seq, these TFs showed an overall down-regulation from 21 to 80DAG, and a good correlation (*r2* = 0.68) between MACE-Seq and dPCR results was obtained (Additional file 3).

The expression of three TFs, VviLBD4, VviHB6, and VviERF3 revealed to be significantly different in between the two heterografts. At 21DAG only VviLBD4 is significantly different between heterografts being down-regulated in the more compatible combination when compared to the less compatible one. At 80DAG, VviLBD4 is more expressed in the more compatible combination, as well as VviHB6 and VviERF3. These TFs are involved in the maintenance of cambium activity, growth, and differentiation. These results showed that the expression of these three TFs is significantly less reduced from 21DAG to 80DAG in the more compatible combination than in the less compatible combination.

### Expression of post-transcriptional regulators at the graft interface

**Fig. 6:**
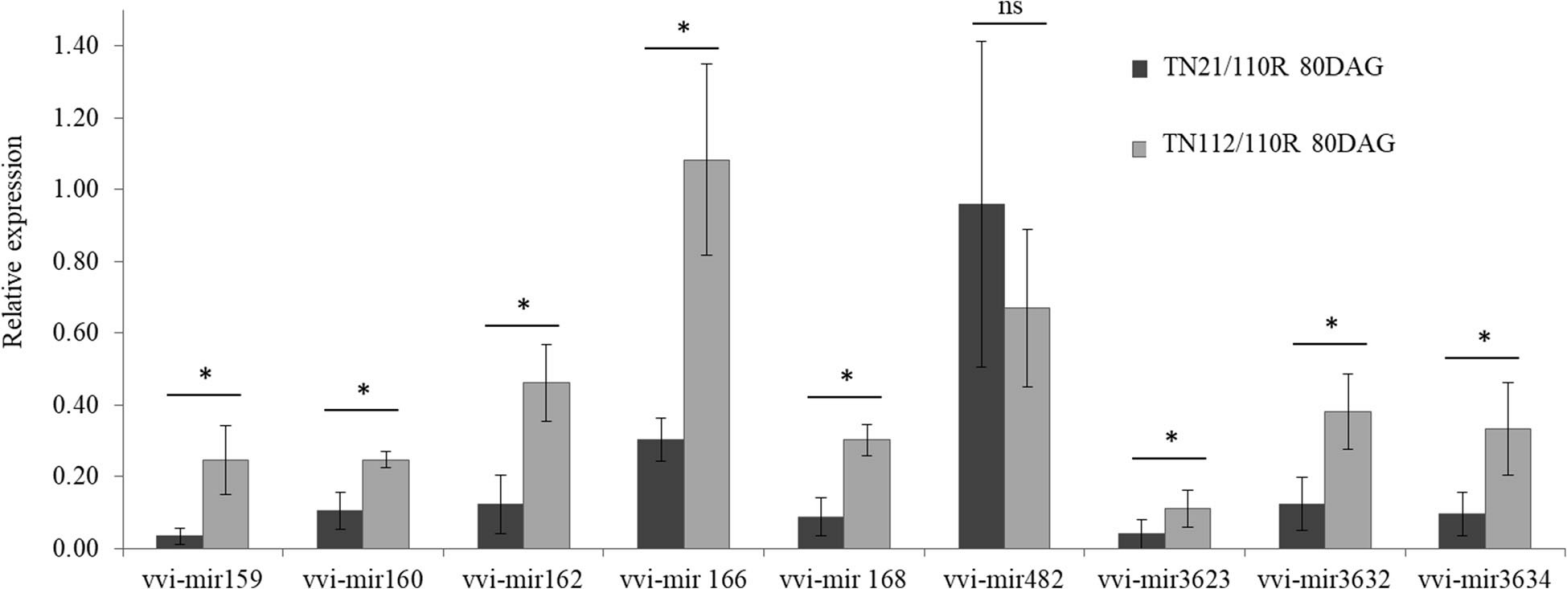
Relative expression of miRNAs measured at 80DAG at the graft union. The expression level was detected by RT-qPCR from tissues collected at the graft union zone of both heterografts. Error bars represent the standard deviation of three biological replications. Asterisk means significant differences with *p* < 0.05 and ‘ns’ non-significant differences, according to one-way ANOVA

microRNA libraries obtained from the total RNA extracted at the graft union tissue were analyzed, and eight miRNAs were selected for expression analysis, based on their abundance in the graft union tissue and in the predicted function of their potential targets (Additional file 4). Quantification by qPCR was performed in the samples collected from the graft zone at 80DAG, since it was the time point when the greatest differences in gene expression between combinations were observed. Results are presented in Fig. 6.

A lower abundance of the majority of the quantified miRNAs in the more compatible heterograft was observed when compared to the less compatible combination. All miRNAs analyzed showed significant differences in the expression between heterografts, except for Vvi-mir482. Vvi-miRNA159 and Vvi-miRNA166 target genes involved in signaling and gene expression regulation. Particularly, Vvi-miRNA166 targets and negatively regulates the expression of TFs of class III Homeodomain leucine zipper (HD-ZIP III), such as REV, PHAB, and homeobox 8 like, known to play an essential role on the regulation of the differentiation on the vascular system [39, 40]. Vvi-miRNA159c targets MYB65 and MYB101 and it has been proposed to be involved in the promotion of PCD and inhibition of growth by reducing cell proliferation [41]. All miRNA predicted targets were checked in the transcriptome data, but low abundance or a false discovery rate (FDR) higher than 0.05 was found.

## Discussion

### TN21 and TN112 have different levels of graft compatibility

The influence of the environment in the construction of a phenotype is observed by the variation of graft success in the 3 years of field trials. Despite this variability in graft success, the TN21/110R combination was more successful than TN112/110R in three independent years. Furthermore, the grafting of these clones in a different rootstock (1103-P) showed the same tendency of graft success. Because the incompatibility of grafting can be defined as failure to form a successful graft union between parts of two plants [7], it is assumed that, in these conditions, the TN21/110R combination is more compatible than TN112/110R.

When comparing DEGs between autografts and heterografts, Cookson et al. [17] observed an up-regulation of plant defense and stress response associated genes at graft interface. That evidence led those authors to suggest that cells at the graft interface can detect the presence of self or non-self-grafting partners. For this reason, in our study, autografts were initially considered as controls for a more compatible graft union. However, our field trials revealed variable autograft successes depending on the genotype, meaning that graft success is genotype dependent even in autografted plants. Only one reference to autograft incompatibility could be found [42] but only for in vitro autografts of *Vicia faba*. Hence, the use of autograft controls must be carefully considered, as the autograft and the heterografts mechanism towards the formation of a well-established graft union may be different. For these reasons, we excluded autografts as controls for the transcriptomic analysis of our assays.

The different behaviour of this clones when autografted or heterografted suggests a rootstock dependent behaviour towards graft success. Besides, the more compatible behaviour of TN21 and the less compatible behaviour of TN112 were both observed in two different rootstocks (110R and 1103-P). It should be noted that both rootstocks are American hybrids of *Vitis berlandieri* x *Vitis rupestris*. If the different levels of compatibility observed are specific to this hybrid species remains to be addressed.

### Compatibility seems to be related with the activation of signaling in an early graft stage

The role of endogenous hormones and other signaling molecules have been described to be important in regulating the interaction between scion and rootstock towards graft union success in *Arabidopsis* [8, 43, 44]. In grapevine, Cookson et al. [16] associated graft union formation at 3 and 28DAG with an up-regulation of genes of cell wall synthesis, secondary metabolism, and signaling. In the present study, a greater abundance of transcripts related to the category “Signaling” (hormone or pathway signaling) was observed earlier (at 21DAG) in the most compatible combination when compared to the less compatible one. Particularly, the role of auxin and ethylene in these two time points should be further investigated. A higher and more precocious abundance of signaling-related transcripts may be interpreted as a greater potential for communication between the graft and the rootstock at an early stage of grafting. This supports the hypothesis that a higher communication potential early in graft union might contribute to graft union success.

### Genes encoding oxidative stress and wound healing proteins are more expressed in the less compatible heterograft at 80DAG

Stilbene biosynthesis is regulated by many different abiotic and biotic stresses [45] and wounding is known to activate the biosynthesis of stilbenes in grapevine berry skin [46], peanut leaves [47] and Scots pine stems [48]. Recently the phenolic analysis of both TN21/110R and TN112/110R combinations showed a significant decrease in phenolic antioxidants sooner in the more compatible one [14]. The higher abundance of transcripts of stilbene synthase, resveratrol synthase, and transcripts of oxidative stress response proteins suggests that the less compatible heterograft has a higher oxidative environment throughout the graft process, hampering a good graft union formation.

At 80DAG, the less compatible combination presents a higher number of transcripts involved in the responses to oxidative stress (polyamine oxidase, glutathione S-transferase, galactinol synthase and peroxidases), and in wound response. In contrast, the more compatible combination presents up-regulated transcripts with important roles in photosynthesis and in cell growth suggesting that growth and development processes are taking place at 80DAG.

At 80DAG the less compatible combination presents a higher accumulation of NAC and WRKY transcription factors, known to be involved in the regulation of necrosis, immune response, and the salicylic acid pathway [49–51]. Furthermore, a transcript encoding a key enzyme in the ABA biosynthesis was found more abundant in the less compatible combination at this stage. This could be interpreted as an increase in stress signaling in the less compatible combination. The importance of the expression of WRKY TFs in grafting has been also suggested by the observation of a significant down-regulation of VviWRKY18, VviWRKY52, and VviWRKY70 in the shoot apical meristem of grapevine heterografts when comparing to autografts [52]. The higher expression of WRKY18 and 70 in the less compatible combination at 80DAG may suggest that the healing process of grafting is was not yet overcome at that time point in that combination.

**Fig. 7:**
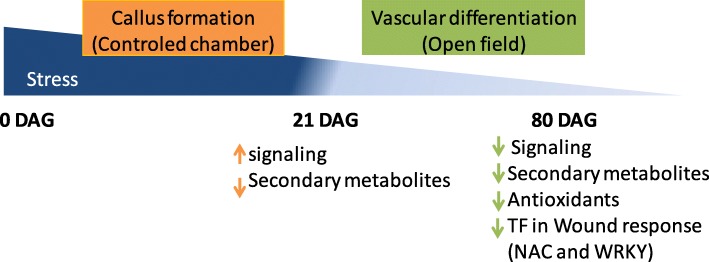
Hypothetical scheme of transcription regulation associated with a compatible graft union. Up arrows indicate more expression, down arrows indicate less expression. Orange arrows refer to the expression at 21DAG and green arrows to the expression at 80DAG

Two of the most highly DEGs, more abundant in the less compatible combination, at 80DAG, are two Arachidonic acid-induced DEA1. DEA1 has been related to the induction of PCD in tomato [53]. Cookson et al. [16] found an up-regulation of a DEA1 gene from 3DAG to 28DAG and suggested a relation with phospholipid signaling processes occurring at the graft interface at this stage (callus induction). According to Cookson’s observation, the higher abundance of this transcript in the less compatible combination at 80DAG (later in graft development) seem to indicate an unfavorable delay in the signaling and in the regulation of PCD processes in the less compatible combination. Altogether, these findings suggest a more stressful environment in the less compatible combination, at 80DAG. This may imply that this combination is unable to deal with the stress resulting from the grafting process in an early stage, being this the possible cause of graft failure. A scheme of the gene expression events that lead to a compatible graft is proposed in Fig. 7.

### A higher expression of TF involved in cambium maintenance and vascular differentiation seems to be important in driving graft success at 80DAG

The importance of a vascular tissue connection to form an effective graft union has been referred by different authors [8, 54–56], although the molecular mechanism and the master regulators involved are still largely unknown. In this study, VviLBD4, a TF involved in the cambium maintenance, was differentially expressed between the more and the less compatible combination at 21 and 80DAG. LBD4 is a member of the Organ boundary (LOB) family and has been associated to cell proliferation, mainly in phloem development, in regulating anatomic features like the multiseriate rays [28] and to callus maintenance in *Arabidopsis* [57]. Cookson et al. [16] verified that this gene is overexpressed 3DAG and that its expression decreases 28DAG. They propose that in the first stage of graft development, the abundance of this TF could be related to the formation and maintenance of non-differentiated callus cells; and later (at 28DAG), the down-regulation could be related to the formation of a functional graft union [16]. In this study, from 21 to 80DAG an inversion in the expression levels occurred for VviLBD4 between the more and less compatible combination. The significant overexpression of VviLBD4 at 80DAG in the most compatible combination, when compared to the less compatible combination, suggests the need for maintenance of the expression of this gene to achieve a more compatible graft.

From 21 to 80DAG the maintenance of the ethylene response factor ERF3 abundance in the more compatible heterograft (TN21/110R) was also observed, while a significant decrease was observed in the less compatible heterograft. ERF family members are involved in growth, development, and biotic and abiotic stress responses [58, 59]. Brackmann and Greb [58] suggest that the ERF transcription factors promote vascular cell divisions downstream of PXY and WOX4. WOX4 is a conserved TF described to have a significant role in promoting differentiation and/or maintenance of the vascular procambium, the initial cells of the developing vasculature [59]. The significant decrease over time in the abundance of this TF in the less compatible combination may suggest a higher difficulty to maintain the production of cambium cells in this combination.

**Fig. 8:**
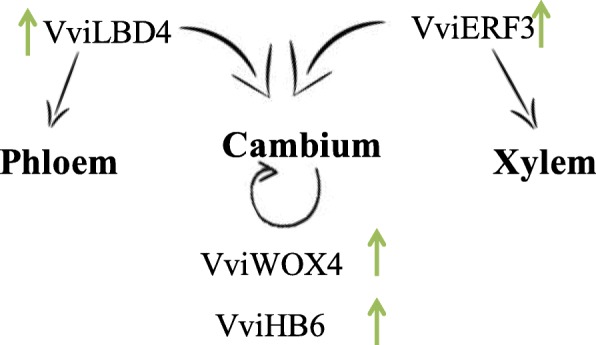
Hypothetical scheme of the transcriptional regulation of vascular differentiation in the mediation of compatible grafts. Putative role of VviLBD4, VviERF3, VviWOX4 and VviHB6 in the vascular differentiation of a more compatible graft when compared to a less compatible one. Arrows indicate significantly different up-regulation in the more compatible heterograft when compared to the less compatible one, at 80DAG

The HB6 TF belongs to HD ZIP I family and negatively regulates responses to ABA [60] ATHB6 was also implicated in cell division and differentiation in developing organs [27]. Although no difference in the expression of VviHB6 at 21DAG was detected between the two heterografts, at 80DAG the higher expression in the less compatible combination may indicate a lower control over ABA signaling and consequently an increase in growth inhibition. In sum, the maintenance along time of adequate expression of these TFs seems to be crucial for a compatible graft union. Based on the expression variation of these TF and their function described in literature, a working scheme of the transcriptional regulation of vascular differentiation in the mediation of compatible grafts is presented in Fig. 8. The role of these TFs in graft union is further supported by Melnyk et al. [61], where these four TFs are associated to graft by being over expressed in *Arabidopsis* grafts when compared to non-grafts and non-graft cuts.

Concerning the post-transcriptional regulation of vascular differentiation, except for Vvi-mir482, all the tested miRNAs are more expressed in the less compatible combination at 80DAG. From the tested microRNAs, Vvi-mir159 and Vvi-mir166 target TFs associated with vascular tissue differentiation. Mir159 targets GAMYB TFs that represses MYB33 and MYB65 in vegetative tissues. Deregulation of these TFs inhibits growth by reducing cell proliferation. More recently, these TFs have also been related to the regulation of vegetative development [41]. Vvi-miRNA166 targets TFs of the class III Homeodomain leucine zipper (HD-ZIP III) known to play an essential role on the regulation of the differentiation of the vascular tissues [39, 40]. Despite the regulatory function of these potential targets, no statistically significant variations (FDR < 0.05) were observed in the RNAseq data, when looking at the expression in the more and the less compatible combination libraries at 80DAG (Additional file 4). This was also observed in all the predicted targets of the eight microRNAS studied. One possible explanation is the very low abundance of the potential miRNAtargets, as the expression of the 58 TF within the potential targets of these eight microRNAs has a median value in the more compatible combination of 59.42 normalized counts, while the less compatible has a value of 57.57 normalized counts (Additional file 4).

## Conclusion

We aimed to understand what drives grapevine graft compatibility in order to find master regulators of graft union. The ultimate goal is to use those molecules as useful markers for early prediction of graft success. For that, we compared heterografts from two clones of the same variety, both grafted to commercial rootstock 110R in a nursery environment, at phenotypic, transcriptional and post-transcriptional levels.

Graft compatibility seems to be driven by increased expression of signaling genes of the metabolic and hormonal pathways, which occurs in the more compatible combination earlier than the less compatible combination, in the callus formation phase. This can be interpreted as having the more compatible combination a greater potential of communication between scion and rootstock. Also, as a result of less oxidative stress, the more compatible combination shows reduced expression of genes of the phenol metabolism and oxidative stress responses.

The less compatible combination has a delay and a higher rate of expression of genes related to wound response and oxidative stress which shows that it deals inefficiently with the establishment of a suitable interaction.

The 80DAG stage seems to be the more adequate stage to evaluate compatibility. In fact, the higher expression of TFs related to cambium maintenance and vascular tissues differentiation and proliferation, as well as, the lower levels of post-transcriptional regulation of TFs also involved in the same processes, clearly characterize the more compatible combination. In this context, VviLBD4, VviERF3, VviHB6 and Vvi-mir159, 160 and 166 could be used, at the 80DAG, as expression markers of a compatible grafting.

## Methods

### Plant material

Two registered clones of the Portuguese cultivar Touriga Nacional (TN), the clone 21 ISA-PT (TN21) of PORVID and the clone 112 JBP-PT (TN112) of JBP/Plansel (https://www.vinetowinecircle.com/castas_post/touriga-nacional/), were grafted on the rootstock 110R (110 Richter - *V. berlandieri* x *V. ruprestis*) and on the rootstock 1103-P (1103 Paulsen – *V. berlandieri* x *V. rupestris*). These grafts resulted in TN21/110R, TN112/110R, TN21/1103-P and TN112/1103-P heterografts. Autografts of the TN21, TN112, and 110R were used resulting in TN21/TN21, TN112/TN112 and 110R/110R.

All grafts and field trials were performed at Plansel nursery located in Montemor-o-Novo, Portugal (38°39′N and 8°13′W). In 2012 trial, 300 TN heterografts in 110R rootstock were established to collect samples for gene expression analysis and to assess the graft successful rate of the two clones of TN. To evaluate the graft success rate of the heterografts in different years and to add the above mention autograft controls, the trial was repeated with 200 grafts in 2015 and 2017. Furthermore, to confirm the behavior of the TN clones in a different rootstock, in the 2017 trial, 100 grafts between the two clones of TN and the rootstock 1103-P were also performed.

### Grafting procedure

All grafting procedures were executed at the Plansel Nursery, as normally performed for commercial purposes. Briefly, hardwood cuttings of the plant material mentioned above were collected in the winter and preserved at 4 °C until grafting. Just before grafting, one-bud cuttings were made for scions and 35 cm cuttings, with nodes disbudded, were used as rootstock. Scions and rootstocks pairs were bench grafted using the omega graft technique. The grafts were dipped in paraffin, supplied with 8-quinolinol (0.11%) and 2,5-dichlorobenzoic acid (0.004%), at 75–80 °C and put in boxes containing peat and transferred to a chamber at approximately 30 °C and 80–90% humidity, for callus induction. After 21DAG combinations were transferred to the field. For each grafting combination three groups of three plants (nine replicates) were harvested in two different time points, 21DAG before being transferred to the field, and 80DAG in field conditions. Only dead plants were excluded from the pools. The pools were made using random but alive plants, i.e. plants with various levels of development. Samples were stored in a refrigerator at − 80 °C until use. From all plants harvested, a longitudinal cut with approximately 1 cm length was made at the graft zone. After removing the bark and the cortex, the remaining tissues (xylem, phloem, and cambium) were scratched and ground in a mortar with liquid nitrogen until a thin powder was obtained and stored at − 80 °C until RNA extraction.

### RNA extraction and purification

Total RNA was extracted using an adaptation of the method described by Chang et al. [62] using 0.1 volumes of NaOAc (3 M, pH 5.2) and two volumes of ethanol in the precipitation steps, to increase precipitation of the microRNAs. For each plant, an independent RNA extraction was performed.

Total RNA concentration and purity were verified using a NanoDrop spectrophotometer ND-2000C (Thermo Scientific) by measuring absorbance at 260/280 and 260/230 and the integrity by electrophoresis on 2% agarose gel (0.5x TBE, Syber safe, Invitrogen). All samples were treated with Turbo DNA-free™ Kit (Ambion, Life Technologies, Ltd.) according to the manufacturer’s instructions. The removal of genomic DNA was screened by PCR, using primers for the intronic region of the gene VIT_18s0001g10160 (Forward-ATAACCTCTCACCACCCAATC and Reverse CTCCAAGATCCCAATCTGTTC). The PCR mixture (final volume of 20 μL) included: 300 ng of total RNA; 3 pmol of each primer; 1x PCR buffer (5X Green GoTaq® Reaction Buffer); 2.5 mM of MgCl_2_, 2.5 mM of dNTPs mix; and 0.5 U of GoTaq® DNA Polymerase. The amplification conditions were the following: an initial denaturing step at 94 °C for 2 min, followed by 30 cycles of 1 min at 94 °C, 1 min at 55 °C and 2 min at 72 °C, with a final extension at 72 °C for 2 min. The amplified gene products were visualized after electrophoresis on a 1% agarose gel (0.5x TBE, Syber safe, Invitrogen). From the nine independent extractions for each experimental condition, total RNA from three samples was pooled with equal quantities, enabling to have three pooled biological replicates.

### mRNA sequencing and expression profiling

Pools of the total RNA extracted from the heterografts, at the two time points sampled, were sent in biological triplicate for deep sequencing by Massive Analysis of 3′-cDNA Ends (MACE) by GenXPro GmbH (Frankfurt Main, Germany). Twelve different libraries were obtained, three biological replicates per each experimental, TN21/110R_21DAG; TN21/110R_80DAG; TN112/110R_21DAG; TN112/110R_80DAG. Libraries were constructed and analyzed by GenXPro GmbH as described by Zawada et al. (2014). The raw sequencing data of the 12 libraries were deposit in NCBI under the project PRJNA517111 and it is available in this address http://www.ncbi.nlm.nih.gov/bioproject/517111. TrueQuant technology was used to remove duplicate reads from the raw dataset, low-quality sequence bases were trimmed and the poly-(A)-tail was clipped off. The barcoded samples were sequenced in an Illumina Hiseq2000, with 1 × 100 bps. MACE reads were mapped onto the *Vitis vinifera* 12X genome assembly. The number of transcripts per gene was normalized by the library size of mapped reads multiplied by 1 million. The resulting contigs of the assembly were annotated by BLASTX to the Swiss-Prot database, CRIBI V1 annotation. The normalization and the analysis of the differentially expressed genes (DEG) were done with DEGSeq R/Bioconductor package [63]. The analysis was performed between heterografts at each time point and between time-points (from 21DAG to 80DAG), for each heterograft separately. Genes were considered significantly differentially expressed with an FDR value < 0.05 and a log2 fold change threshold of *x* ≥ |1|. Only transcripts with more than 100 counts at least in one of the condition were considered. Functional characterization was performed using the MapMan web tool Mercator (http://www.plabipd.de/portal/mercator-sequence-annotation). Protein sequences of all DEG were obtained using BioMart in Phytozome v.12 (https://phytozome.jgi.doe.gov/) to create a mapping file for Mercator. Functional annotation obtained was crossed with the one published in Grimplet et al. [26]. An additional excel file containing the DEG between heterografts and between timepoints is provided (Additional file 5). The functional categories of the DEG from 21 to 80DAG were tested for significance using the PageMan enrichment analysis applying the Fisher test [64].

Heatmaps were constructed using Heatmapper online tool (http://www.heatmapper.ca/expression/). Clustering of the DEG between time-points was performed based on Kendall’s Tau distance and average linkage, with normalized counts of the single libraries of the genes DE.

### microRNA sequencing

One pooled sample per condition was sent for miRNAs sequencing on an Illumina Hiseq2000 run by Fasteris (Geneve, Switzerland).

Polyacrylamide gel was used to select and purify small RNAs from 18 to 30 nucleotids (nt) followed by the bound of 3p and 5p adapters. Subsequently, cDNA was synthesized and amplified generating the libraries for Illumina sequencing. Low-quality reads (FASTq value < 13) were removed and adaptor sequences were trimmed, using the Genome Analyzer Pipeline (Fasteris) as described in Galli et al. [65] Sequences shorter than 18 nt and longer than 25 nt were excluded from further analysis. To identify phylogenetically conserved miRNAs, sRNA sequences were mapped to a set of all conserved non-redundant Viridiplantae obtained from miRBase database (Release 19, August 2012) using Bowtie v 0.12.7. The frequency of identified miRNAs was obtained by aligning the conserved precursors identified in this study and the sRNA library using Bowtie v 0.12.7 with the default parameters. For target prediction, psRNA target database (http://plantgrn.noble.org/psRNATarget/) and literature review were used.

### qPCR

Three independent samples of each experimental condition were used for cDNA synthesis according to the manufacturer’s instructions of qScript™ microRNA cDNA Synthesis Kit (Quanta, Bioscience). Briefly, miRNAs were first polyadenylated in a poly(A) polymerase reaction and then qScript Reverse Transcriptase was used to convert the poly(A) tailed miRNAs into cDNA using an oligo-dT adapter primer. The qPCR amplification reactions were performed using the PerfeCTa Universal PCR Primer (specific to the unique sequence of the oligo-dT adapter primer) and the PerfeCTa® SYBR® Green SuperMix for iQ™, as forward primers the exact mature miRNA sequences were used (Additional file 6). The reaction conditions were performed according to the qScript™ microRNA Quantification System, with a final volume of 20 μL, 5 ng of total RNA equivalent was used in each reaction and the 3-step cycling protocol was used to improve specificity. Reactions were performed in an iQTM 5 Real-Time PCR Detection System (BioRad, Munich, Germany), and melting curve analysis was performed to ensure the specificity of primers. Primers efficiency was determined using the LinRegPCR program [66].

The expression levels of miRNAs were normalized to small interfering RNA 4 (siRNA4) and siRNA41 using the Pfaffl eq. (1 + Efficency)^−ΔΔCt^ method and the autograft 110R as a control sample (Pfaffl, 2001). The two reference genes selected, were pre-screened for their expression and the expression of 5.8 s rRNA, were used as a reference gene in *Vitis* miRNA expression analysis in Wang et al. [67]. After analysing their variance between samples using geNorm and NormFinder [68] in the Genex software (MultiD, Goteborg, Sweden) the siRNA4 and siRNA41 of *Medicago truncatula* recently discovered and published by Formey et al. [69], revealed to be the best reference genes. Statistical analysis was performed with Statistica software (Statsoft Inc., Tulsa, USA). To evaluate the variance between combinations, one-way analysis of variance (ANOVA) was performed (*p* < 0.05).

### Digital PCR

QuantStudio™ 3D digital PCR (QS3D) System (Life Technologies) was used for the absolute quantification of TFs expression. Three biological samples at 21 and 80DAG cDNA was synthesized using 200 ng of total RNA previously treated by RNase-free DNase and then reverse transcribed using ImProm-II™ Reverse Transcription System following the manufacturer’s instructions. The dPCR reaction solutions were prepared with 20 ng of cDNA, the QS3D reaction mix, 0,9 μM of primer forward and reverse and 0,25 μM of each FAM and VIC labeled probes (Additional file 7). Digital PCR was performed according to Santos et al. (2017). Data analysis and management were performed using QuantStudio™ 3D Analysis Suite™ software (https://apps.lifetechnologies.com/quantstudio3d/). Confidence level was set to 95% and desired precision to 10%, in the Poisson Plus algorithm version 4.4.10. Further statistical analysis was performed with Statistica software (Statsoft Inc., Tulsa, USA). After confirming the non-normal distribution using Shapiro-Wilk test, the Wilcoxon-Mann-Whitney test (non-parametric) was applied to the dPCR data, evaluating the variation between time points and between combinations.

## Additional Files

Additional file 1: Unsuccessful grafts detected throughout the field trial in the heterografts (A) and autografts (B). Percentage of unsuccessful grafts detected until the 80DAG are represented in blue, the ones detected only at the end of cycle are represented in red. The percentage is calculated in relation to the total number of grafts. The success grafts are represented in green. (DOCX 19 kb)

Additional file 2: Heatmap of transcripts involved in hormone signaling, pathway signaling, regulation of gene expression (TF) and cell wall regulation. The transcripts were found DE between time points (21DAG and 80DAG), specifically DE in both combinations. A total of 108 DEGs in TN21/110R (A) and a total of 90 DEG in TN112/110R (B) are shown in all four libraries separately. The transcripts are organized by functional annotation. The asterix highlights the combination in which the transcripts are statistically DE (FDR < 0.05). (DOCX 624 kb)

Additional file 3: Correlation between the TFs analyzed by MACE-Seq and digital PCR. (DOCX 16 kb)

Additional file 4: MicroRNAs selected for qPCR analysis, their annotated potential targets, and respective functions. (XLSX 75 kb)

Additional file 5: List of DEGs between combinations and between time points. (XLSX 592 kb)

Additional file 6: Primer sequences of Vvi- microRNAs used for expression quantification by qPCR. (DOCX 14 kb)

Additional file 7: Primers and TaqMan®-Probes sequences used for gene expression quantification by dPCR. (DOCX 15 kb)

## Abbreviations

ABA: Abscisic acid
DAG: Days after grafting
DEG: Differentially expressed genes
dPCR: Digital PCR
IAA: Auxin
miRNA: Microrna
qPCR: Quantitative PCR
TF(s): Transcription factor(s)
TN112: Touriga Nacional clone 112
TN112/110R: TN112 grafted in the rootstock 110R
TN21: Touriga Nacional clone 21
TN21/110R: TN21 grafted in the rootstock 110R
