# Supplementary material for: Understanding the molecular mechanisms underlying graft success in grapevine
Source: BMC Plant Biol. 2019 Sep 11;19:396. doi: 10.1186/s12870-019-1967-8 (PMC6737599; doi:10.1186/s12870-019-1967-8)
Supplement: Supplementary file 1 — Unsuccessful grafts detected throughout the field trial in the heterografts (A) and autografts (B). Percentage of unsuccessful grafts detected until the 80DAG are represented in blue, the ones detected only at the end of cycle are represented in red. The percentage is calculated in relation to the total number of grafts. The success grafts are represented in green. (DOCX 19 kb) [file 12870_2019_1967_MOESM1_ESM.docx]

**B**

**Fig. 3- Unsuccessful grafts detected throughout the field trial in the heterografts (A) and autografts (B).** Percentage of unsuccessful grafts detected until the 80DAG are represented in blue, the ones detected only at the end of cycle are represented in red. The percentage is calculated in relation to the total number of grafts. The success grafts are represented in green.
