# Supplementary figures and images for: Understanding the molecular mechanisms underlying graft success in grapevine
Source: BMC Plant Biol. 2019 Sep 11;19:396. doi: 10.1186/s12870-019-1967-8 (PMC6737599; doi:10.1186/s12870-019-1967-8)

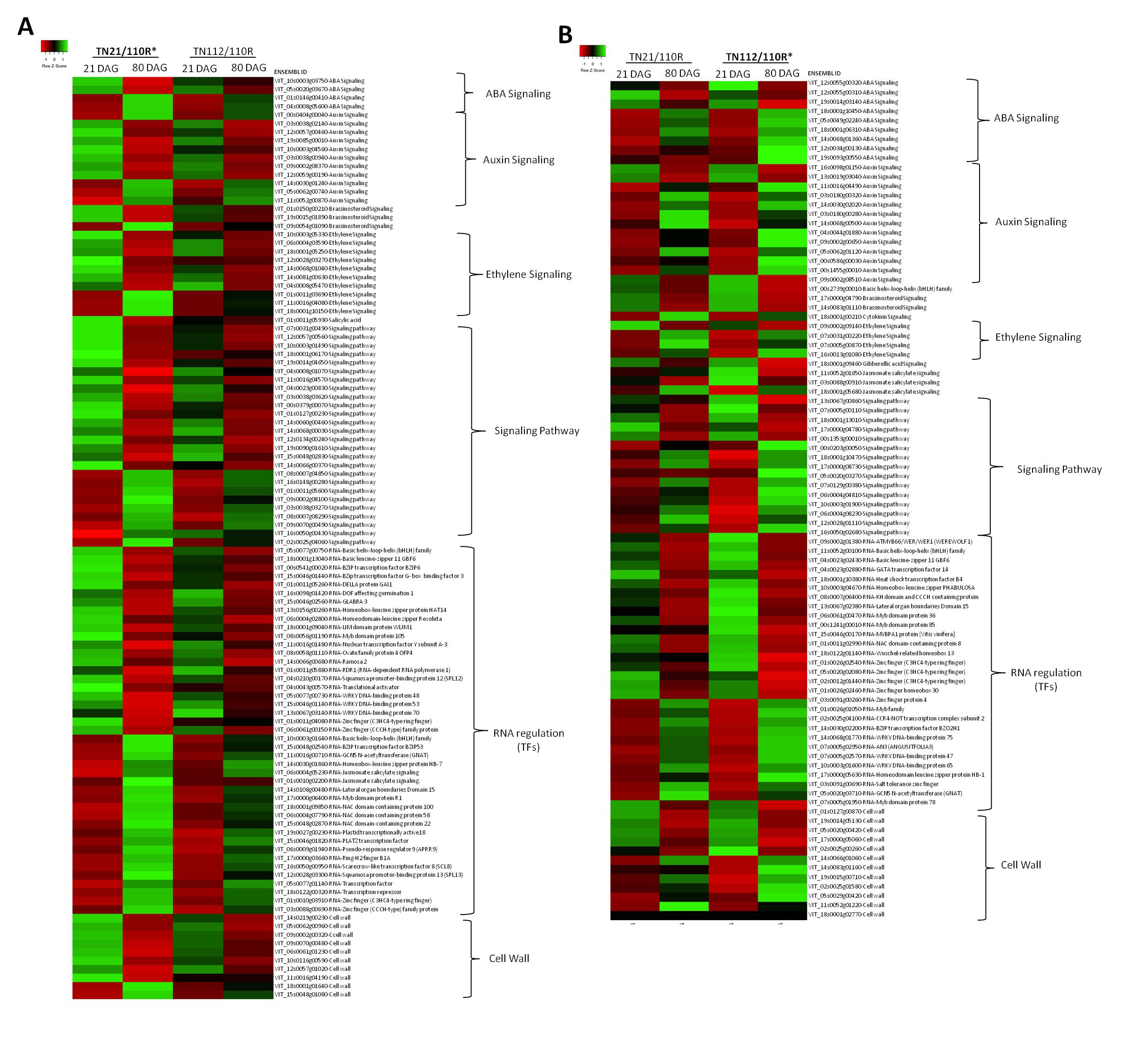


Additional file 2-

Supplement: Supplementary file 2 — Heatmap of transcripts involved in hormone signaling, pathway signaling, regulation of gene expression (TF) and cell wall regulation. The transcripts were found DE between time points (21DAG and 80DAG), specifically DE in both combinations. A total of 108 DEGs in TN21/110R (A) and a total of 90 DEG in TN112/110R (B) are shown in all four libraries separately. The transcripts are organized by functional annotation. The asterix highlights the combination in which the transcripts are statistically DE (FDR < 0.05). (DOCX 624 kb) [file 12870_2019_1967_MOESM2_ESM.docx]
