# Supplementary material for: Understanding the molecular mechanisms underlying graft success in grapevine
Source: BMC Plant Biol. 2019 Sep 11;19:396. doi: 10.1186/s12870-019-1967-8 (PMC6737599; doi:10.1186/s12870-019-1967-8)
Supplement: Supplementary file 3 — Correlation between the TFs analyzed by MACE-Seq and digital PCR. (DOCX 16 kb) [file 12870_2019_1967_MOESM3_ESM.docx]

**Additional file 3.** Correlation between the TFs analyzed by MACE and digital PCR.
