# Supplementary material for: Understanding the molecular mechanisms underlying graft success in grapevine
Source: BMC Plant Biol. 2019 Sep 11;19:396. doi: 10.1186/s12870-019-1967-8 (PMC6737599; doi:10.1186/s12870-019-1967-8)
Supplement: Supplementary file 6 — Primer sequences of Vvi- microRNAs used for expression quantification by qPCR. (DOCX 14 kb) [file 12870_2019_1967_MOESM6_ESM.docx]

| **Additional file 6.** Primer sequences of Vvi- microRNAs used for expression quantification by qPCR | | |
| --- | --- | --- |
| Vvi-microRNAs | Fw Primers for real-time PCR (5’→3’) | Length (nt) |
| Vvi-miR159c | TTTGGATTGAAGGGAGCTCTA | 21 |
| Vvi-miR160c | TGCCTGGCTCCCTGTATGCCA | 21 |
| Vvi-miR166 a/c/e/f | TCGATAAACCTCTGCATCCAG | 21 |
| Vvi-miR168 | TCGCTTGGTGCAGGTCGGGAA | 21 |
| Vvi-miR482 | TCTTTCCTACTCCTCCCATTCC | 22 |
| Vvi-miR3623-3p | TCACAAGTTCATCCAAGCACCA | 22 |
| Vvi-miR3632-3p | TTTCCCAGACCCCCAATACCAA | 22 |
| Vvi-miR3634 | TTTCCGACTCGCACTCATGCCGT | 23 |
| siRNA4 | AACCGCGGAAACCGAAAAGCTC | 22 |
| siRNA41 | ACCGCGGAAACCGAAAAGCTC | 21 |
| 5.8s rRNA | ACGTCTGCCTGGGTGTCAC | 19 |
