# Supplementary material for: Understanding the molecular mechanisms underlying graft success in grapevine
Source: BMC Plant Biol. 2019 Sep 11;19:396. doi: 10.1186/s12870-019-1967-8 (PMC6737599; doi:10.1186/s12870-019-1967-8)
Supplement: Supplementary file 7 — Primers and TaqMan®-Probes sequences used for gene expression quantification by dPCR. (DOCX 15 kb) [file 12870_2019_1967_MOESM7_ESM.docx]

**Additional file 7:** Primers and TaqMan®-Probes sequences used for used for gene expression quantification by dPCR.

| **Gene Id (gene name)** | **Forward primer sequence** | **Reverse primer sequence** | **Probe sequence** | **Dye** |
| --- | --- | --- | --- | --- |
| \| VIT_04s0008g06000 (Ethylene-responsive transciption factor 3 - ERF003) \| \| --- \| | AAGTTCAAGTGGACAGCAACA | CATAGTCAAGCAACTCCTCAATC | CCCAGCAGTTCAAGCCTCTTGAAG | VIC |
| VIT_18s0001g02540 (Auxin responsive regulator protein 9 - ARR9) | TGGAGAAGCTGCTCACTGTT | ATCATTGTCTCGGCCATCTA | TCATGTGACTTGCGTGGAATCTGG | VIC |
| VIT_06s0004g03130 (Auxin response factor protein 4 – ARF4) | CTGCAGCTGGCTGTAAACTG | TGCCTTGCTTGTGAACCTTA | TCCTTGACTGGAGAAACTCCTCCAAA | VIC |
| VIT_07s0141g00290 (Auxin responsive protein 16 - IAA16) | CATGGAAGTTGCCCGGAAA | TCACCACCTCACTTCCTTCA | AAGCCCGACCTGAACTTGGAGGCGACGGA | VIC |
| VIT_19s0027g01120 (Lateral organ boundaries protein 4 – LBD4) | GGACTGTGTCTTTGCTCCTTAC | CATCTTATTGACATTGCTTGCT | TTTCCGGCTGATGAGCCACATAAG | FAM |
| VIT_18s0001g10160 (Wuschel homeobox protein 4 – WOX4) | AAGCTTGGATGCTCTGATGA | AGCATCTCCAAGATCCCAAT | AGGTGGGACGCGGTGGAATC | FAM |
| VIT_18s0001g06430 (Homeobox-leucine zipper protein 6 – ATHB-6) | TGATGTCTCCAGCGTCTTCT | GATGCTGAAACGCTTTACTT | TGTGCTTCTTTGTCATCACCTTCACCA | FAM |
| VIT_16s0013g00890 (Ethylene responsive factor – ERF1A) | AGCGTTAGCTATCAGGGAAA | CCTACAGCAGAAATGGAATGA | TGAAGCCTGCCCAAAGTGAGAAGA | FAM |
